# Acrodermatitis Enteropathica: A Case Report

**DOI:** 10.1097/MD.0000000000003553

**Published:** 2016-05-20

**Authors:** Nicolai Nistor, Lavinia Ciontu, Otilia-Elena Frasinariu, Vasile Valeriu Lupu, Ancuta Ignat, Violeta Streanga

**Affiliations:** From the Pediatrics Department (NN, O-EF, VVL, AI, VS), “Gr. T. Popa” University of Medicine and Pharmacy; and “St Mary” Children Emergency Hospital (LC), Iasi, Romania.

## Abstract

Acrodermatitis enteropathica is a rare genetic autosomal recessive disorder, characterized by periorificial dermatitis, alopecia, and diarrhea. It is caused by mutations in the gene that encodes a membrane protein that binds zinc. We report a 14-month-old boy, admitted for erythematous, scaly and pustular lesions, initially located in the inguinal and perianal regions and on thighs, and very few erythematous lesions on the face. Due to the numerous bacterial skin superinfections with *Staphylococcus aureus*, including abscesses that required surgical incision, the clinical picture was modified, leading to a delayed establishment of the diagnosis. Later, the symptoms became suggestive for this disease, the diagnostic having been confirmed by low plasma zinc values. Under zinc therapy, skin lesions improved significantly in a few days, with favorable outcome. Two months later, the skin lesions almost disappeared.

Abscesses due to bacterial skin superinfections may lead to initially misdiagnosed acrodermatitis enteropathica.

## INTRODUCTION

Acrodermatitis enteropathica (AE) is an autosomal recessive condition resulting in severe zinc deficiency. The deficiency is caused by a defect of dietary zinc absorption in the duodenum and jejunum.^1^ Zinc is an essential co-enzyme in metal enzymes (like alkaline phosphatase); it is an important structural component of gene regulatory proteins (required, for example, for the intracellular binding of tyrosine kinase to T-cell receptors) and it has also a function in regulation (has the ability to regulate gene expression). Consequently, there are multiple signs and symptoms in severe deficiency: growth retardation, impaired immune function, and multiple skin or gastrointestinal lesions.^2^ AE usually presents itself during childhood after weaning. Symptoms vary with age. Signs and symptoms in infancy can include diarrhea, mood changes, anorexia, and neurological disturbance. In schoolchildren and toddlers, zinc deficiency is characterized by growth retardation, alopecia, weight loss, and recurrent infections. Spontaneous remission may occur in teens.^3^

Laboratory diagnosis is hazardous, zinc levels in serum, urine, or hair are used (although they are not specific, neither sensitive), zinc absorption tests are cumbersome, genetic testing is definite (defect in 8q24, gene SLC39A4) though not routinely available. Most clinicians therefore depend on immediate results of a therapeutic zinc regimen (3–30 μmol/kg body weight).

In Denmark, 1:500000 people are affected by AE.^4^ Another study reported also an incidence of 1 per 500,000 children without predilection for race or sex.^5^

## CASE PRESENTATION

We present the case of a Caucasian 14-month-old boy, admitted for skin lesions that occurred from the first months of life. The boy was born prematurely, weighing 1800 g, without any perinatal medical history. He had been breastfed for 1 month and then formula-fed up to 5 months, followed by diversified nourishment. His family history revealed an apparently healthy 5-year-old sister and another sister who died at the age of 5 months with similar superinfected skin lesions. His parents stated that the skin lesions started at the age of 2 months, about 1 month after the infant was weaned from breastfeeding; initially, lesions were discrete and partially ameliorated with local treatment. At the age of 10 months, the boy had bacterial skin superinfections with *Staphylococcus aureus*, including suprapubic and right thigh abscesses, which required surgical incisions followed by antibiotic treatment with clindamycin, intravenous, for 10 days, according to sensitivity testing. The antibiotic treatment for *S aureus* determined the disappearance of the pus, but the lesions of AE persisted. His body weight was 8000 g when he was 10 months old. He was born weighing 1800 g, so he had shown no evidence of delay in his growth.

**FIGURE 1:**
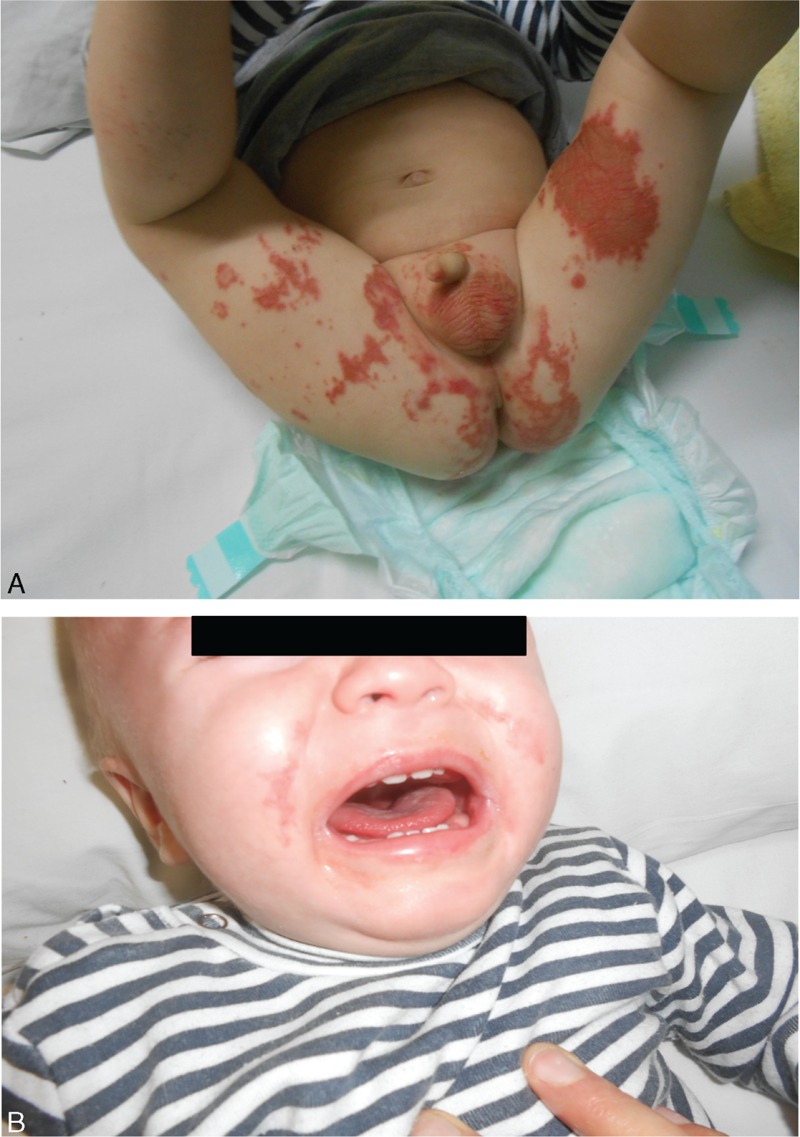
Skin lesions in the diaper area, thighs (A), and face (B).

**FIGURE 2:**
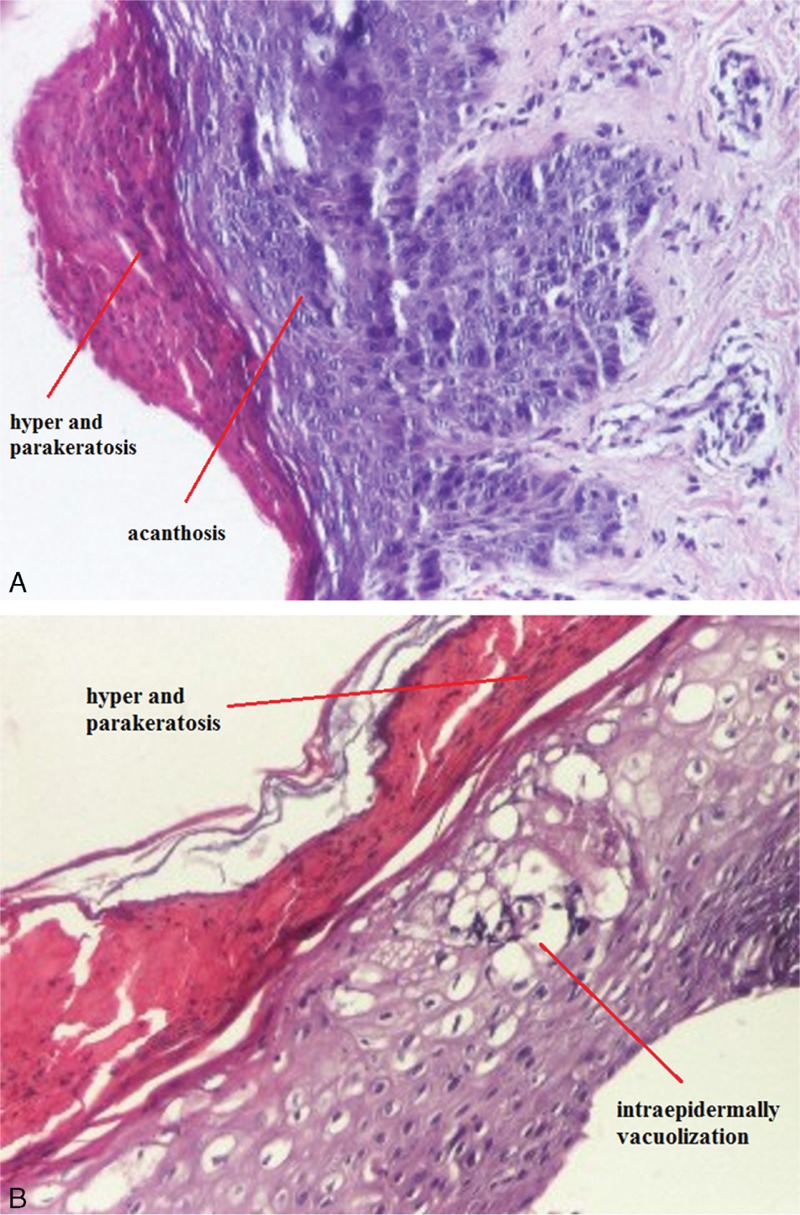
Epidermal hyperplasia with acanthosis, hyper and parakeratosis, crusts, HE ×40 (A) and intraepidermally vacuolization, HE ×100 (B).

At admission to our clinic, the patient was in relatively poor condition. Informed consent was obtained from the caregiver each time the patient was hospitalized. Skin examination revealed erythematous and micro pustular lesions predominantly on the buttocks, thighs, and perioral region (Figure 1). The bacteriological examination of the pustules content showed *Escherichia coli*. Antibiotic treatment with piperacillin-tazobactam, intravenous, was conducted under sensitivity testing with slight improvement in skin lesions. The dermatologist suspected napkin psoriasis, and a local treatment with fluticasone propionate was started. Skin biopsy was performed, and then, the patient was discharged to continue local treatment until the arrival of the skin biopsy result. Histopathological examination of left thigh skin biopsy revealed epidermal hyperplasia with acanthosis, clustered necrotic keratinocytes and parakeratosis, crusts, and intraepidermal vacuolization (Figure 2).

**FIGURE 3:**
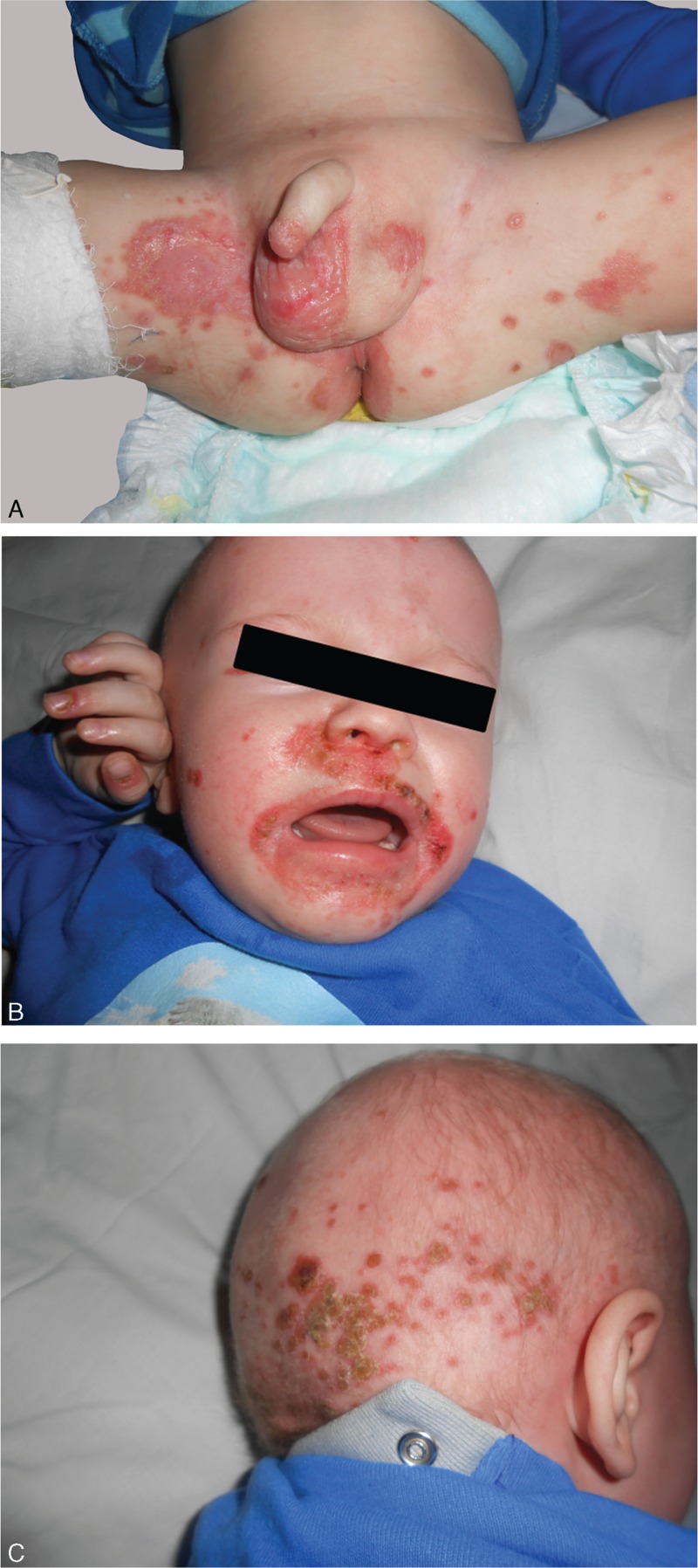
Erythematous, scaly, and pustular lesions located in the inguinal and perianal regions, on the thighs (A), periorificial on the face, perionychia (B), and on the scalp with alopecia areas (C).

One month later, he was readmitted with more extensive erythematous, scaly and pustular lesions located in the inguinal and perianal regions, on the thighs, on the scalp, and periorificial on the face. The hair was thin, with areas of alopecia, the eyebrows and the eyelashes were fallen, perionychia was present (Figure 3). We noticed intermittent episodes of psychomotor agitation, but he never presented with diarrhea. An abscess was developed on the right thigh, which required surgical incision, followed by antibiotic treatment with clindamycin, according to sensitivity testing for *S aureus* isolated from pus.

We considered a diagnosis of AE. The patient's serum and urinary zinc levels were low: 23 μg/dL (normal value = 65–118 μg/dL), respectively, 11.4 mg in the urine of 24 hours (normal value = 150–1200 mg/24 h), thereby confirming the diagnosis. Serum alkaline phosphatases, a zinc-dependent enzyme, was also to the lower limit of normal (50 U/L, normal = 40–600 U/L). His immune function revealed IgA and IgG deficiency (IgA = 10.87 mg/dL, IgG = 426.29 mg/dL). HIV test results were negative. Other causes of malabsorption were excluded: antitissue transglutaminase antibodies (IgA and IgG) for celiac disease and β-lactoglobulin for cow's milk protein intolerance were negative. Also, iontophoresis was negative (chloride concentration was 34 mmol/L).

Zinc therapy was instituted at a dosage of 3 mg/kg daily of elemental zinc, with rapidly favorable evolution: the lesions became smaller and paler after 2 to 3 days of treatment. The infant was discharged to continue daily zinc treatment; the doses were to be adapted to the evolution and weight.

**FIGURE 4:**
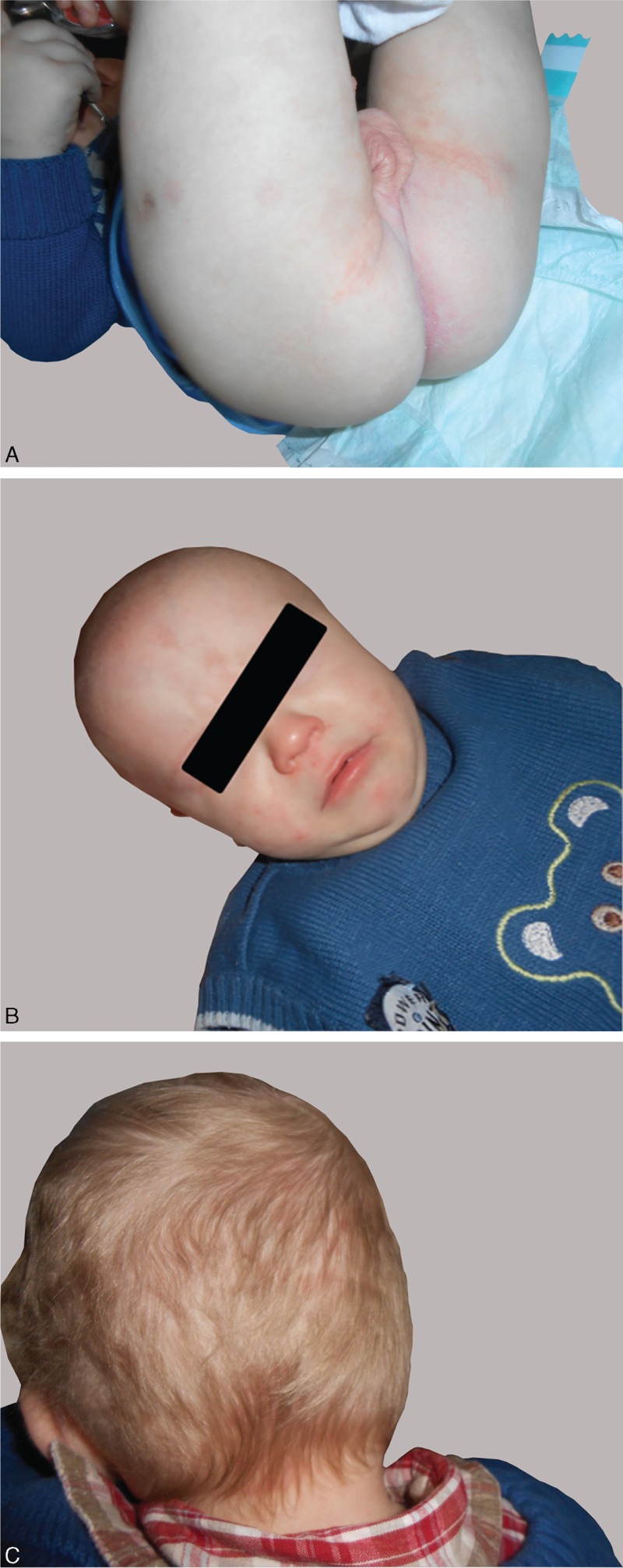
Cutaneous aspects after 2 months of treatment, with remission of periorificial lesions and alopecia.

On 2 months’ check-up, the skin lesions and alopecia were almost completely remitted (Figure 4), the agitation episodes disappeared, and the child had a normal behavior. He is currently continuing his zinc treatment, and he will be undergoing regular check-ups.

## DISCUSSION

Zinc is an essential trace element required for the proper functioning of all cells, and plays an important role in the metabolism of protein, carbohydrate, and vitamin A. It is a cofactor of numerous metal enzymes such as alkaline phosphatases, alcohol dehydrogenase, RNA polymerase, and numerous digestive enzymes.^2^ Zinc deficiency can be acquired or inherited. There are many causes of acquired zinc deficiency: premature infants, low birth weight, zinc deficiency in maternal milk, exclusive parenteral nutrition, malabsorption syndromes such as Crohn disease and celiac disease, alcoholism, low calcium and phytate diet, and kwashiorkor.^6^

The hereditary deficiency of zinc is classically known as “acrodermatitis enteropathica.” It is caused by an autosomal recessive mutation of SLC39A4 (solute carrier family 39 member 4) gene on chromosome 8q24.3, which determines a congenital partial or total deficiency of the zinc transporter protein zinc-ligand binding protein 4 (ZIP 4).^7^

The clinical manifestations of acquired zinc deficiency and AE are similar and consist of 3 essential symptoms: periorificial dermatitis, alopecia, and diarrhea. This clinical triad is complete in only 20% of patients with AE.^3^ The disease begins with symmetrical erythematous, squamous or eczematous lesions, sometimes vesiculobullous or pustular lesions, located around perioral, anogenital, and acral areas. The severity of the skin lesions is variable. Without treatment, skin damage becomes erosive and spreads to other periorificial areas of the face (eyes, nose, and ears), neck, lower abdomen, back, inguinal area, and thighs. In some instances, the skin lesions may appear psoriasiform. Other mucous and cutaneous signs consist of diffuse alopecia, loss of eyelashes and eyebrows, glossitis, gingivitis, stomatitis, onychodystrophy, onycholysis, and pachyonychia.^6,8^ Diarrhea is the predominant extracutaneous symptom. Unfortunately, it is also the most variable, and it can be intermittent or totally absent. In children with watery diarrhea, general symptoms and neuropsychological disorders are common (irritability, lethargy, depression, anorexia), and also growth retardation, weight loss, anemia, and ophthalmic involvement (photophobia, blepharitis, conjunctivitis). Secondary bacterial infections (with Gram-positive and sometimes Gram-negative) or candidiasis (*Candida albicans*) are common and they can modify the clinical picture.^9^ So, clinical findings in AE described in the literature encompass a broad spectrum, making the diagnosis challenging.

In our patient, repetitive bacterial skin superinfections with *E coli* and *S aureus* modified the symptomatology, also requiring surgery, which leaded to a diagnosis delay.

The diagnosis is based on clinical symptoms and is confirmed by low plasma zinc levels and rapid clinical response to zinc supplementation.

In our case, in the absence of certainty diagnostic and with periorificial lesions that seemed to be typical for AE, we performed zinc test. Low serum alkaline phosphatase value, a zinc-dependent metal protease, has an indirect role in establishing the diagnosis.^2^ Histopathologic findings have a less significant contribution at establishing the diagnosis, because microscopic aspects are not specific, but, in our case, we performed biopsies in the absence of certainty diagnostic. Histopathology of cutaneous lesions reveals a psoriasiform necrolysis, most often pale, by cytoplasmic vacuolization, subsequent focal, or confluent necrosis of keratinocytes in the superficial part of the epidermis and parakeratosis more or less confluent. In resolving or chronic AE lesions, psoriasiform hyperplasia is present.^2,10^

Acrodermatitis enteropathica has been described in breast-fed infants related to low zinc levels in the mothers.^10,11^ In our case, the skin lesions started at the age of 2 months, about 1 month after the infant was weaned from breastfeeding.

Serum alkaline phosphatase levels, in our child, were to the lower limit of normal (50 U/L). In another study also, the authors reported a low value (18 U/L) at the time of diagnosis.^11^

The differential diagnoses to consider are psoriasis, atopic dermatitis, seborrheic dermatitis, contact dermatitis, skin infections with candida, Langerhans cell histiocytosis, and cystic fibrosis.^12^ Once the disease has been diagnosed, the treatment consists of oral zinc supplementation given daily, which leads to a dramatic disappearance of the symptoms within a few days. With treatment, the survival rate is 100%.^6^ AE must be treated and monitored throughout the entire life.

The limitation of our case report is the fact that on presentation to a genetic consult, DNA samples were taken to carry out specific genetic tests for both the patient and his parents, but we still could not perform them, because these techniques are currently unavailable in Romania.

## CONCLUSIONS

Acrodermatitis enteropathica is a rare condition; early recognition of cutaneous manifestation being necessary, particularly because of immunodeficiency and infection concerns in these patients. Patients with repetitive bacterial skin superinfections may be misdiagnosed for months after initial presentation, as in the case of our patient. In the presented case, the diagnosis was delayed because of change in clinical symptoms, through the absence of the diarrhea and through the recurrent bacterial superinfection. Anyway, in the end, the evolution under treatment was favorable.
